# The Misuse of Hospitals and Honorary Medical Service

**Published:** 1895-07-13

**Authors:** 


### The Hospital

An Institutional Journal of

The Medical Sciences and Hospital Administration, with

AN EXTRA NURSING SUPPLEMENT.

Vol. XVIII.—No. 459.] [July 13, 1895.

### The Misuse of Hospitals and Honorary Medical Service.

The authenticated figures published in our Hospital Sunday Supplement of June 15th last have already excited great interest in the minds of the public, and are likely to engage the attention of the medical profession for some months to come. Including the endowed hospitals, one million six hundred and ninety-five thousand two hundred and seventy-six actual patients found their way to the hospitals and medical institutions of London in 1893, all of whom received some kind of treatment. This is a startling fact; but the figures are even more striking when they are examined a little in detail and compared with those during previous decades.

If we confine the figures to the voluntary hospitals alone, it appears that nine hundred and sixty-six thousand five hundred and twenty-eight in and out patients were treated by hospitals receiving grants from the Metropolitan Hospital Sunday Fund in 1893. Comparing the growth of hospital patients with the population of London, we find the increase to have been as follows: The number of patients and the population increased in 1883, as compared with 1873, to an almost identical extent, namely, by 18 and 16 per cent. respectively. Ten years later, however—that is at the end of 1893—while the population had increased as compared with 1883 by only 10 per cent., the number of patients treated had increased by nearly 50 per cent. Further, if we compare the figures for 1893 with those for 1873, we find that whereas in twenty years the population had increased by about 28 per cent., the number of patients relieved by the hospitals had increased by a little over 75 per cent. In other words, whereas in 1873 and 1883 one out of every six of the population received medical relief from the voluntary hospitals, in 1893 nearly one out of every four of the population became a patient at one or more of these institutions. The *Provincial Medical Journal*, in a long article based upon our Hospital Sunday Supplement, regrets that we did not dwell upon the object lesson they presented to the medical profession. Our answer is simple. Our immediate object was to help the hospitals to secure an additional income of £25,000, and we felt the best way to secure this would be to state the facts in as telling a manner as possible, with the view of arousing and fixing public attention. We are glad to see that our effort has succeeded, and that there is good reason to hope that at least £50,000 will be raised through the Metropolitan Hospital Sunday Fund this year. This being the case, we are now at liberty to consider what lessons the figures have for the profession and the public.

The first lesson undoubtedly is that the system of admitting patients to the metropolitan hospitals requires reform and amendment. No one who has studied the question and taken care to ascertain the classes which at present supply patients to the hospitals can doubt that a vast number of them ought not to receive free medical relief. It follows that the present uncontrolled system of free admission does much injustice to the whole body of the medical profession, and that it tends to demoralise the patients. So far as the hospital managers are concerned, it must be remembered that they are not free agents, for they have to administer a system as they received it from their predecessors. This system is based upon two principles antagonistic to each other and harmful to the profession and the hospitals. The first is that the medical staff shall render honorary medical service; the second that the committees cannot have a free hand to enforce economy and institute checks upon the admission of patients which are based upon business considerations alone. It follows that whereas eleemosynary medical service entails grievous hardship upon the younger members of the medical profession, it also makes it impossible to administer our hospitals upon sound business principles, by which alone economy can be secured and abuse prevented. Hence it will be necessary to abolish, or at any rate to greatly lessen, the amount of honorary medical service at present required from the medical profession in justice to medical practitioners and the hospitals. Once abolish eleemosynary medical service, and the committees of management will be free to institute regulations and adopt a system which will remedy hospital abuse, and reduce expenditure to an appreciable extent.

We have not space to day to go fully into the questions thus raised. So far as the medical profession is concerned, the enormous and unjustifiable growth in the number of patients treated in the large hospitals tends to place the general practitioners in large towns in antagonism to the consultants or honorary medical officers of the hospitals. The latter, who are unpaid, hold that they must be left free to attend all patients who are sent to them, whilst the general practitioners rightly insist that uncontrolled free relief is rendering it impossible for the ablest doctors to maintain themselves in the lower middle class and poorer portions of a city like London. These questions must be faced and determined by the medical profession as a whole. The general practitioners have the remedy in their own hands. Every one of them has paid large fees in order to learn his profession, and he has a claim upon the medical school of his choice, which he can make effective by combination. If the whole of the general practitioners who were students, say at Guy's, or St. Thomas's, or the London Hospital, were to act together and make a unanimous representation to the honorary medical staff of the great hospital to which their school is attached, there can be no doubt that a change would speedily be effected. This is the first step to be taken by those members of the medical profession who realise the dangers of the present situation and are determined to remove them in the best interests of all concerned. There are other and even larger questions involved which we must leave for a future opportunity.

